# P01 Small study, big improvements: assessing the impact of halving the administration time of high dose ceftriaxone in a tertiary centre OPAT clinic

**DOI:** 10.1093/jacamr/dlaf230.008

**Published:** 2025-12-04

**Authors:** Lottie Platel

**Affiliations:** Greater Glasgow & Clyde Health Board, Glasgow, Scotland; Glasgow School of Medicine & Dentistry, Glasgow, Scotland

## Abstract

**Background:**

Prior to 2023, there was conflicting guidance around the ideal administration time for high dose ceftriaxone (4 g). Given this lack of clarity, we found that some Health Boards in Scotland were administering it over 30 min, whilst others were giving it over 60. In a previous study, we used analysis of OPAT patient data collected from the Queen Elizabeth’s University Hospital (QEUH) and Forth Valley Royal Hospital (FVRH) OPAT departments to prove that there was no significant difference in frequency of side effects and/ or treatment failures when high dose ceftriaxone was administered over 30 min, as opposed to 60 (RR 0.1 in favour of 30 min administration time, *P*=0.07).

**Objectives:**

The objectives of this follow-up study were to identify: (i) hours of OPAT clinic time saved; (ii) money saved as a result of additional treatment slots opened; and (iii) qualitative patient feedback.

**Methods:**

We assessed a period of 130 days between January (when the intervention was introduced) and May 2025 to identify any patients requiring higher dose ceftriaxone and the number of days of treatment over this period. As a single slot for these patients used to take 60 min and now takes 30; each patient visit to OPAT represented 30 min of time saved. We subsequently used the Scottish Health Technologies Group OPAT Calculator and accompanying support documents to calculate the cost-saving impact this intervention would have on the Hospital (SGHT, 2021).

**Results:**

During this time we treated 17 patients with high dose ceftriaxone. Treating these patients required 105 × 30 min slots in our OPAT clinic. As administration time has been halved, we saved 52.5 h of OPAT time over this period. Extrapolating this to 1 year, we predict we will have saved 142 h of OPAT time annually. Our calculations show that by freeing up hundreds of 30 min slots in our OPAT clinic, we are saving the hospital in the region of £87 000 per year. In terms of patient experience; the change to service means less patient time will be taken up in clinic receiving infusions as well as time saved for any friends or family transporting patients to and from clinic.

**Conclusions:**

The original study was relatively straightforward and conducted by a single staff member however, the time and cost savings were substantial. The study demonstrates how even a small-scale Quality Improvement Project, particularly when instituted within a cost-saving service such as OPAT, can have a big impact.

